# Efficacy and safety of serplulimab in solid tumors: a meta-analysis

**DOI:** 10.3389/fphar.2025.1604874

**Published:** 2025-06-18

**Authors:** Peimeng Shen, Tao Zhang, Lina Hao, Ming Jing, Yanxin Wu, Shuwen Yu

**Affiliations:** ^1^ School of Pharmaceutical Sciences, Shandong University, Jinan, Shandong, China; ^2^ Department of Pharmacy, Shandong Electric Power Central Hospital, Jinan, Shandong, China; ^3^ Department of Pharmacy, Children’s Hospital Affiliated to Shandong University, Jinan, Shandong, China; ^4^ Phase I Clinical Trial Center, Qilu Hospital of Shandong University, Jinan, Shandong, China; ^5^ NMPA Key Laboratory for Clinical Research and Evaluation of Innovative Drug, Shandong University, Jinan, Shandong, China

**Keywords:** safety, meta-analysis, solid tumors, serplulimab, efficacy

## Abstract

**Objective:**

The goal of this study was to investigate the effectiveness and safety of serplulimab in advanced solid tumors through a meta-analysis approach.

**Methods:**

An electronic search was conducted across the Embase, Web of Science, PubMed, and Cochrane Library databases, covering the period from each database’s inception through 6 May 2025. Meta-analysis and related analyses, including subgroup, sensitivity, and publication bias assessments, were performed using Stata 16.0. The Cochrane Risk of Bias Assessment Tool (version 5.1.0) was utilized to measure the quality of randomized controlled trials (RCTs). For single-arm studies, quality was evaluated using the Methodological Index for Non-Randomized Studies (MINORS).

**Results:**

Ten studies, including three RCTs and seven single-arm studies, were analyzed, involving 2,020 patients. In the analysis of RCTs, serplulimab significantly elevated overall survival (OS) [HR = 0.68, 95% CI: 0.59–0.79, P < 0.01], disease control rate (DCR) [RR = 1.04, 95% CI: 1.01–1.08, P < 0.05], progression-free survival (PFS) [HR = 0.53, 95% CI: 0.47–0.61, P < 0.01], and objective response rate (ORR) [RR = 1.30, 95% CI: 1.09–1.56, P < 0.01]. The analysis of single-arm studies revealed that the ORR for serplulimab in solid tumors was [ES = 45%, 95% CI: 31%–59%, P < 0.01], and the DCR was [ES = 71%, 95% CI: 63%–80%, P < 0.01]. Among the ten studies, the most common adverse events included reductions in platelet count (0.32, 95% CI: 0.20–0.43), white blood cell count (0.30, 95% CI: 0.17–0.44), anemia (0.29, 95% CI: 0.09–0.48), and proteinuria (0.28, 95% CI: 0.17–0.38).

**Conclusion:**

Based on current research, serplulimab appears to be effective for solid tumors. However, given the limitations of the studies, for example, possible selection bias in single-arm studies, further multicenter, high-quality, large-sample RCTs are necessary to validate this conclusion.

## 1 Introduction

Currently, the treatment options for malignant tumors are diverse, including surgical resection, radiation therapy, and pharmacological therapy. Surgical resection is invasive, with a long recovery period, and cannot cure metastatic tumors. Radiation therapy may damage surrounding healthy tissues, and some tumors are either resistant to radiation or develop resistance over time. In terms of pharmacological treatments, both traditional chemotherapy drugs and novel antitumor agents are used. Traditional chemotherapy drugs include alkylating agents, antimetabolites, antitumor antibiotics, and plant-based drugs. While traditional chemotherapy drugs inhibit cancer cells, they also suppress normal tissue cells, leading to side effects such as hair loss, vomiting, and pain, which significantly impact patients’ daily lives.

Among the newly approved antitumor drugs, the most notable are immune checkpoint inhibitors (ICIs), along with a range of tyrosine kinase inhibitors (small molecules) and monoclonal antibody-targeted drugs (large molecules). The rapid advances in ICIs have contributed to a pivotal shift in cancer treatment strategies. Inhibitors of Programmed death-1 (PD-1), along with a ligand PD-L1 and CTLA-4 (cytotoxic T-lymphocyte antigen 4) have become standard treatment options for many malignant tumors (Koseki et al., 2024; Ribas and Wolchok, 2018; Tang et al., 2018). Compared with traditional chemotherapy, these drugs targeting the PD-1 pathway can prolong overall survival (OS) and reduce toxicity in various solid tumors (Borghaei et al., 2015; Ferris et al., 2016; Harrington et al., 2023; Fradet et al., 2019; Nishijima et al., 2017). Approved PD-1/PD-L1 inhibitors demonstrate varying levels of efficacy and safety across different diseases (Yi et al., 2022; Passiglia et al., 2018; Xu et al., 2018).

Serplulimab (HLX10) is a humanized IgG4 monoclonal antibody targeting PD-1, demonstrating antitumor effects and manageable adverse effects in several cancers (Qin et al., 2022; Ren et al., 2023; Cheng et al., 2022). In a Phase III trial (Cheng et al., 2022), compared to placebo plus chemotherapy, OS was notably enhanced in advanced small cell lung cancer (SCLC) patients when serplulimab was combined with chemotherapy. Based on these findings, serplulimab has received Orphan Drug status for SCLC in the U.S. (Henlius, 2022a), and has been approved by China’s National Medical Products Administration (NMPA) for treating advanced microsatellite instability-high (MSI-H) solid tumors (Henlius, 2022c). Serplulimab plus carboplatin and albumin-bound paclitaxel is approved for squamous non-small cell lung cancer (NSCLC) (Henlius, 2022b), and serplulimab plus fluorouracil-based and platinum-based therapies is approved for PD-L1-positive metastatic esophageal squamous cell carcinoma (ESCC) (Henlius, 2023).

Several Phase I-III clinical trials on serplulimab for managing various tumor types have been conducted (Qin et al., 2022; Ren et al., 2023; Cheng et al., 2022; An et al., 2023; Song et al., 2023; Zhou et al., 2023; Ho et al., 2024; Wang et al., 2024; Ren et al., 2025; Liu et al., 2024); most of the current studies are single-arm trials; and there remains controversy regarding the efficacy of serplulimab. Therefore, this study employs a meta-analysis approach to combine both single-arm and RCT studies to determine the effectiveness and safety of serplulimab in the management of solid tumors, aiming to resolve these controversies and deliver new evidence for treatment options for cancer patients.

## 2 Materials and methods

The protocol has been registered in the International Prospective Register of Systematic Reviews (PROSPERO: CRD42024593376).

### 2.1 Retrieval strategy

A systemic, computer-based search was conducted in Embase, Web of Science, PubMed, and Cochrane Library databases to identify relevant studies on serplulimab for solid tumors. The search period spanned from each database’s inception to 6 May 2025. Search terms included “Serplulimab,” “Hansizhuang,” “HLX10,” and terms related to neoplasms and cancer, such as “Tumor,” “Neoplasm,” “Cancer,” “Malignancy,” and “Benign Neoplasms.” A detailed search strategy is outlined in Supplementary Material S1.

### 2.2 Inclusion and exclusion criteria

Inclusion criteria: 1) Study types: Clinical studies published domestically or internationally on the use of serplulimab for solid tumors, including single-arm studies and Phase I, II, or III RCTs: 2) Study population: Individuals aged 18 or older with solid tumors, regardless of cancer type or metastatic status: 3) Intervention: Patients receiving serplulimab, either alone or in combination with chemotherapy: 4) Outcome measures: Survival outcomes, including objective response rate (ORR) with complete response (CR) and partial response (PR), disease control rate (DCR), progression-free survival (PFS), and OS, as well as safety outcomes, including therapy-related adverse reactions and the rates of adverse events of all levels, particularly those grade three or higher.

Exclusion criteria: Literature reviews, animal studies, meta-analyses, case reports, duplicate publications, studies without reported relevant outcome data, or studies where necessary data were unavailable.

### 2.3 Literature search and data extraction

Two researchers imported the retrieved literature into EndNote reference management software. Duplicate studies were first excluded, and then studies were screened as per the eligibility criteria. Any conflicts were addressed by seeking input from a third researcher. The subsequent data were collected from the included studies: publication year, tumor type, first author, sample size, average patient age, gender ratio, ECOG performance score, treatment regimen, and outcome measures.

### 2.4 Risk of bias evaluation

The Cochrane Risk of Bias Tool (version 5.1.0) (Higgins et al., 2011)was used by two researchers to examine the reliability of the included RCTs. This tool assesses randomization methods, selective reporting, concealment of allocation, blinding, data integrity, and additional biases. Each aspect was classified as low, high, or unclear risk of bias. For single-arm studies, the MINORS criteria (Slim et al., 2003) were adopted to assess quality, with each criterion rated from 0 to 2, yielding a maximum total score of 16. The assessment included study objectives, patient inclusion consistency, expected sample size, appropriate endpoints reflecting study goals, objective evaluation of outcomes, follow-up duration, dropout rates, and sample size estimation.

### 2.5 Data analysis

Meta-analysis and publication bias tests were conducted using Stata 16.0 statistical software. For RCTs, binary data such as DCR and ORR were analyzed using risk difference (RD) as the effect size (ES). Survival data, including OS and PFS, were analyzed using hazard ratios (HR). ES and 95% confidence intervals (CI) were calculated for single-arm studies. Heterogeneity was assessed with the I^2^ statistic or Cochran’s Q test, where I^2^ values of 0%, 25%, 50%, and 75% indicated no, low, moderate, and high heterogeneity, respectively. A random-effects approach was utilized for I^2^ ≥ 50%, with sensitivity analysis to explore heterogeneity sources. A fixed-effects approach was applied for I^2^ < 50%. Publication bias was evaluated using Egger’s test, with P < 0.05 considered statistically significant.

## 3 Results

### 3.1 Review of literature and study characteristics

**FIGURE 1:**
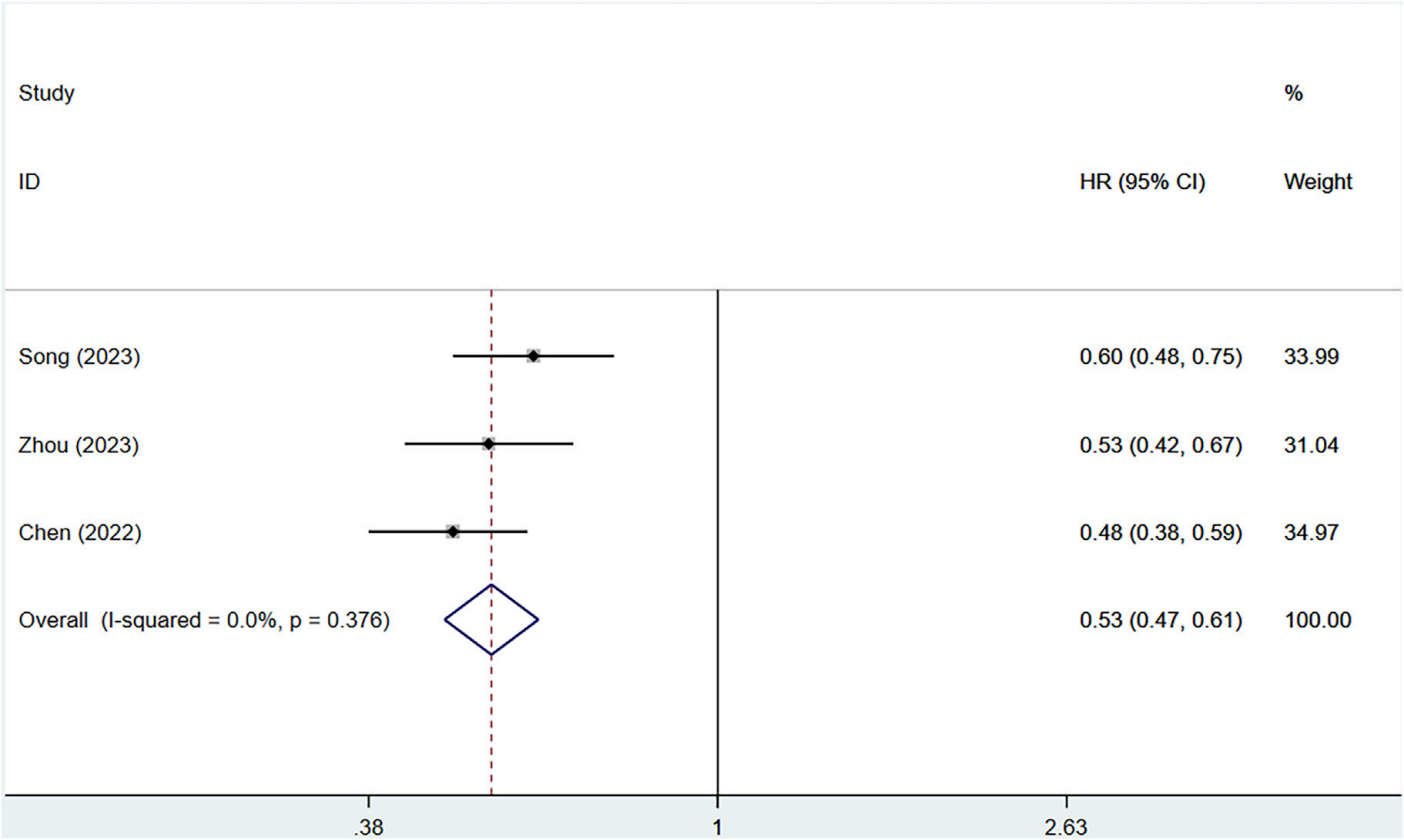
PRISMA flowchart illustrating the study process. PRISMA stands for Preferred Reporting Items for Systematic Reviews and Meta-Analyses.

A total of 530 articles were identified. After duplicates were removed, 199 articles remained. Fifteen articles were shortlisted for full-text review after abstract and title screening, and ultimately, 10 studies were selected for analysis. Among these, three were RCTs (Cheng et al., 2022; Song et al., 2023; Zhou et al., 2023), and six were single-arm studies (Qin et al., 2022; Ren et al., 2023; An et al., 2023; Ho et al., 2024; Wang et al., 2024; Ren et al., 2025; Liu et al., 2024). A total of 2,020 patients participated in the ten studies, with 1,462 and 558 in the experimental and control groups, respectively. The diseases included in the studies were SCLC, NSCLC, ESCC, HCC, metastatic colorectal cancer, cervical cancer. Among these participants, there were 1,668 males and 352 females. The process of selecting literature is depicted in Figure 1. A summary of the key details for the included studies is provided in Table 1. The basic information on the excluded research can be found in Supplementary Material S5.

**TABLE 1:** Basic information of the included studies.

Randomized controlled trial													
Study	Year	Country	Sample size	Mean age	Gender (M/F)	Tumor type	ECOG	Site of metastasis	Intervention	Outcome			
										OS (months)	PFS (months)	ORR	DCR
Chen^[14]^	2022	China	Serplulimab:389 Placebo:196	Serplulimab:63 Placebo:62	481/104	extensive-stage (SCLC)	0/1	Liver Brain	Serplulimab 4.5 mg/kg +Carboplatin AUC 5 +Etoposide 100 mg/m2, first-line Placebo +Carboplatin AUC 5 +Etoposide 100 mg/m2	15.4	5.7	312/370	358/370
Song^[20]^	2023	China	Serplulimab:368 Placebo:183	Serplulimab:64 Placebo:64	470/81	(ESCC)	0/1	Lymph node Long Liver bone	Serplulimab3mg/kg + cisplatin (50mg/m2)+5-fluorouracil (1,200 mg/m2),first-line Placebo + cisplatin (50mg/m2)+5-fluorouracil (1,200 mg/m2)	15.3	5.8	212/368	298/368
Zhou^[21]^	2023	China	Serplulimab:358 Placebo:179	Serplulimab:63 Placebo:63	488/49	Advanced Squamous (NSCLC)	0/1	Liver Brain	serplulimab4.5 mg/kg +nab-paclitaxel (100mg/m2)+carboplatin (5/6 mg/mL/min),first-line Placebonab-paclitaxel (100mg/m2)+carboplatin (5/6 mg/mL/min)	22.7	8.3	215/358	300/358
Single-armed experiment													
Ren^[13]^	2022	China	Group A:20 Group B:21	Group A:52 Group B:53	36/5	Advanced Hepatocellular carcinoma (HCC)	0/1	Extrahepatic metastases	GroupA serplulimab 3 mg/kg + HLX04 5 mg/kg, subsequent-line GroupB serplulimab 3 mg/kg + HLX04 10 mg/kg, subsequent-line	A:11.6 B:14.3	A:2.2 B:NR	A 6/20 B 3/21	A 8/20 B 10/21
Ren^[24]^	2025	China	Group D:61	Group D:55	54/7	advanced hepatocellular carcinoma (HCC)	0/1		GroupD serplulimab 3 mg/kg + HLX04 10 mg/kg, first-line	20.4	7.3	17/58	39/58
Qin^[12]^	2022	China	108	55	55/53	(MSI-H) or (dMMR) tumors	0/1	Colorectal cancer, Endometrial cancer, Gastric cancer, other	Serplulimab 3 mg/kg, once/2W	MEAP:NR SIEAP:NR	MEAP:NR SIEAP:4.2	MEAP:26/68 SIEAP:13/42	MEAP:46/68 SIEAP:23/42
Ho^[22]^	2024	Taiwan	29	60	16/13	histologically confirmed, measurable or evaluable advanced or metastatic solid tumors	0/1/2	Lung Colon Tonsil Esophagus Other	0.3 mg/kg (n = 3) 1.0 mg/kg (n = 4) 3.0 mg/kg (n = 6) 10.0 mg/kg (n = 16)	NR	3.5	2/25	15/25
An^[19]^	2023	China	21	50.8	0/21	Cervical cancer	0/1	Lung, Distant lymph node	Serplulimab 4.5 mg/kg, once/3W	15.5	5.7	12/21	16/21
Wang^[23]^	2024		57	61	44/13	metastatic colorectal cancer	0/1	Liver Lung	Serplulimab300 mg+ HLX04 (7.5 mg/kg)+HLEOX	NR	17.2	36/55	47/55
Liu^[25]^	2024	China	30	64.5	24/6	esophageal squamous cell carcinoma (ESCC)	0/1	lymph node、liver、lung、Bone	Serplulimab200 mg+ HLX041000 mg,once/2W	NR	7.8	18/30	21/30

### 3.2 Quality assessment of the literature

**FIGURE 2:**
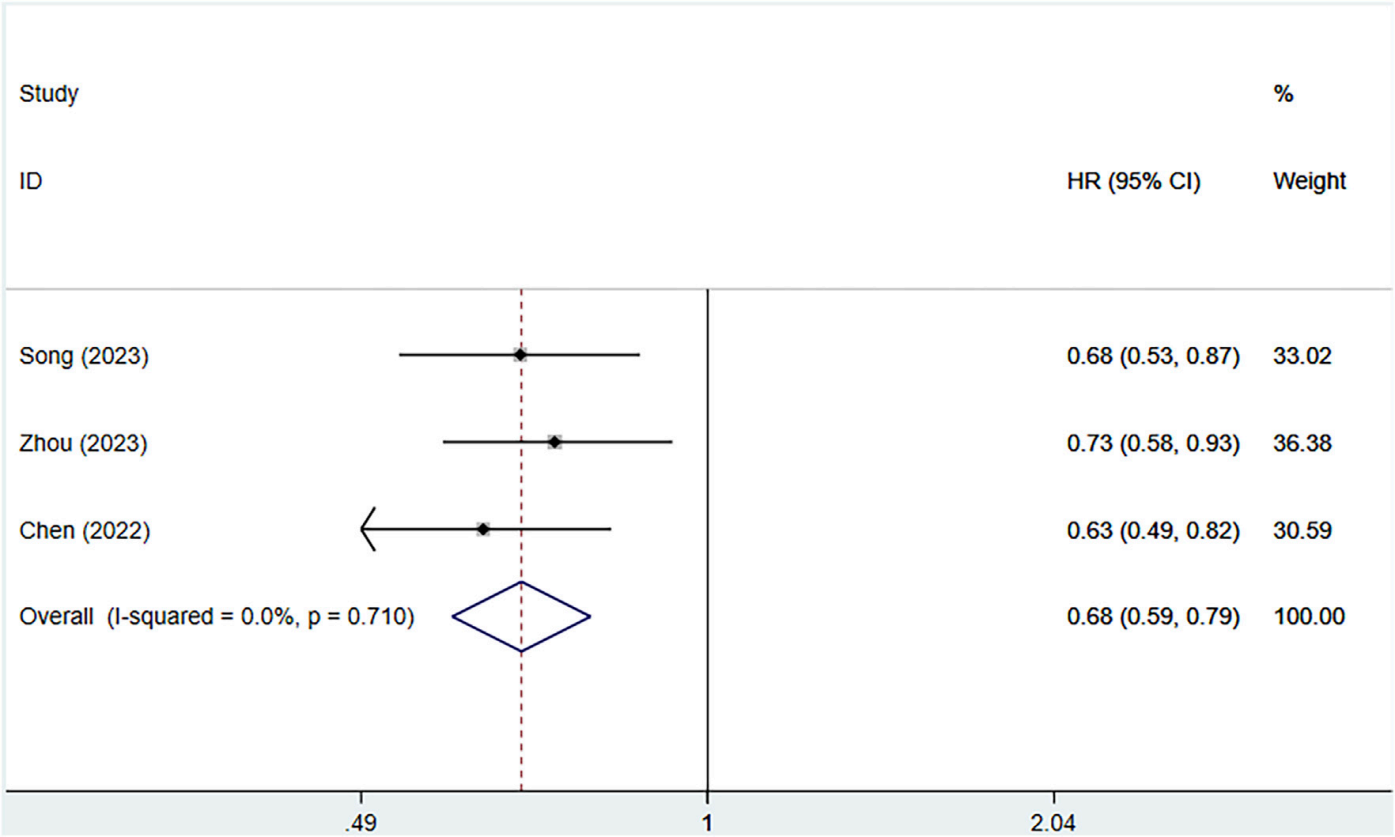
Risk of bias assessment for RCT studies.

Three RCTs (Cheng et al., 2022; Song et al., 2023; Zhou et al., 2023) were measured for quality utilizing the Cochrane Risk of Bias tool, with results summarized in Figure 2. Three studies (Cheng et al., 2022; Song et al., 2023; Zhou et al., 2023) described its random sequence generation process, concealment of allocation, and blinding was applied to both participants and researchers. Two studies (Cheng et al., 2022; Song et al., 2023) used blinding during outcome assessment. No selective reporting was observed, and all studies had complete outcome data. Additionally, no other sources of bias were identified, as shown in Figure 2. For the seven single-arm studies (Qin et al., 2022; Ren et al., 2023; An et al., 2023; Ho et al., 2024; Wang et al., 2024; Ren et al., 2025; Liu et al., 2024), the MINORS scores were ≥12 points. All 7studies (Qin et al., 2022; Ren et al., 2023; An et al., 2023; Ho et al., 2024; Wang et al., 2024; Ren et al., 2025; Liu et al., 2024) clearly stated their research objectives, and the included patients were consistent with the inclusion criteria. The data collected were in accordance with the research protocol established prior to the study. The follow-up duration in all studies was sufficient, and the loss to follow-up rate was below 5%. Two studies (Qin et al., 2022; Ren et al., 2023) estimated the sample size. These details are summarized in Table 2.

**TABLE 2:** MINORS score for single-arm studies.

Study	Year	Clear statement of the study objective	Consistency in patient inclusion	Collection of data as per the predefined plan	Appropriateness of endpoints in reflecting the study objective	Objectivity in the assessment of endpoints	Adequacy of follow-up duration	Loss to follow-up rate below 5%	Estimation of sample size	Total score
An	2023	2	2	2	2	0	2	2	0	12
Ren	2022	2	2	2	2	0	2	2	0	12
Qin	2022	2	2	2	2	0	2	2	2	14
Ho	2024	2	2	2	2	0	2	2	0	12
Wang	2025	2	2	2	2	0	2	2	2	12
Ren	2025	2	2	2	2	0	2	2	0	14
liu	2024	2	2	2	2	0	2	2	0	12

### 3.3 Meta-analysis results of RCT studies

#### 3.3.1 Objective response rate (ORR)

**FIGURE 3:**
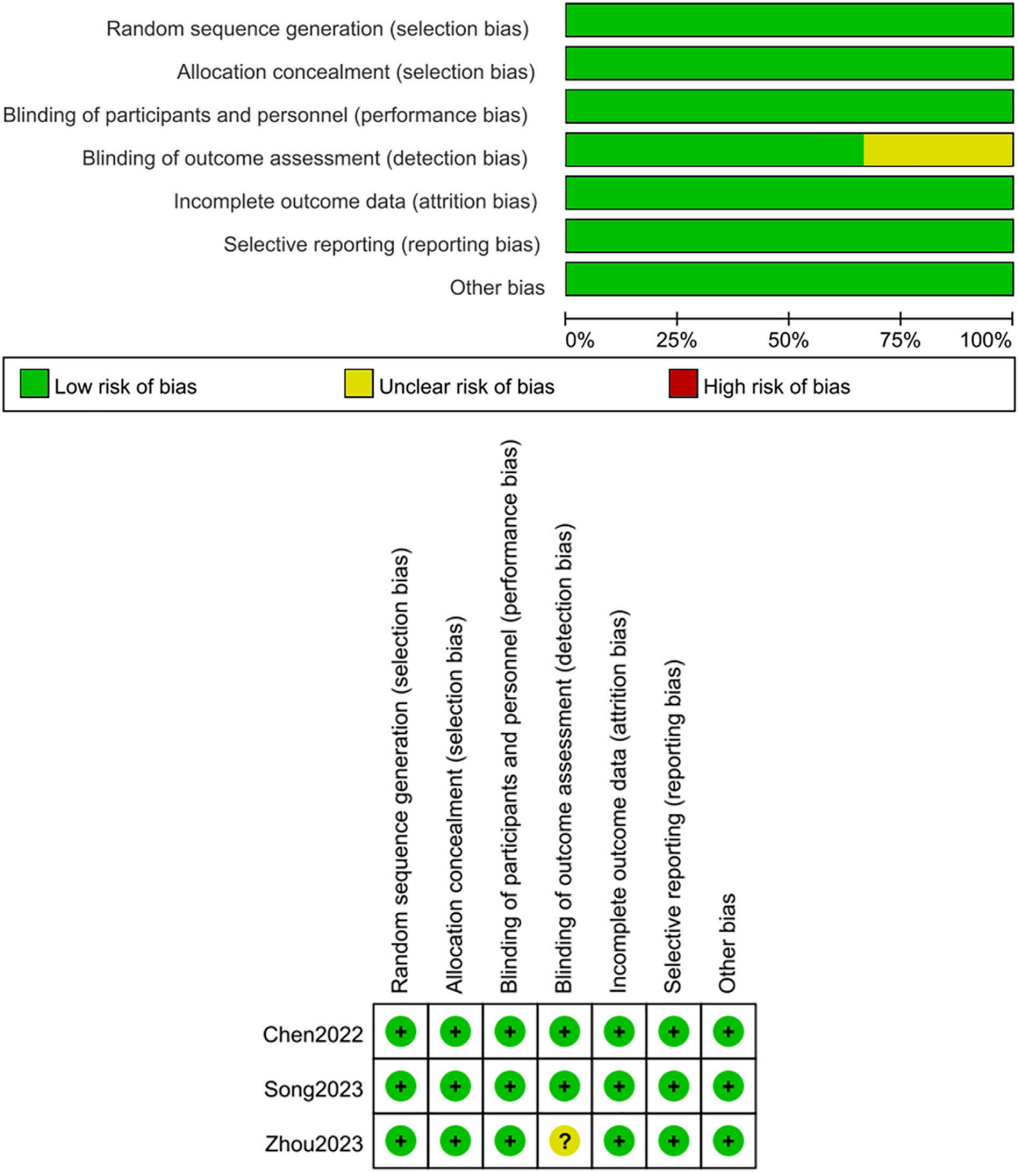
Forest plot of the meta-analysis of ORR in RCT studies.

Three studies, comprising a total of 1,673 patients, assessed the DCR and ORR of serplulimab in late-stage solid tumors, including SCLC, NSCLC, and ESCC. Among these, 1,115 and 558 patients were in the serplulimab and placebo groups, respectively. With significant heterogeneity (I^2^ = 73.4%, P = 0.023), a random-effects model was utilized. Serplulimab significantly improved the ORR in patients with solid tumors [RD = 0.15, 95% CI (0.10–0.19), P < 0.01], as illustrated in Figure 3. Heterogeneity was low (I^2^ = 29.6%, p = 0.241), supporting a fixed-effects model. Sensitivity analysis of the three studies showed low sensitivity, confirming the stability of the results, as shown in Supplementary Figure S1. A P-value >0.05 was observed in Egger’s test for publication bias, indicating a low likelihood of publication bias, as presented in Supplementary Figure S4. The combined analysis of three results showed that the absolute difference in ORR of Serplulimab in combination with chemotherapy ranged from 9.8% to 19.9%, suggesting that it has a consistent effect of remission rate enhancement in a variety of solid tumors.

#### 3.3.2 Disease control rate (DCR)

**FIGURE 4:**
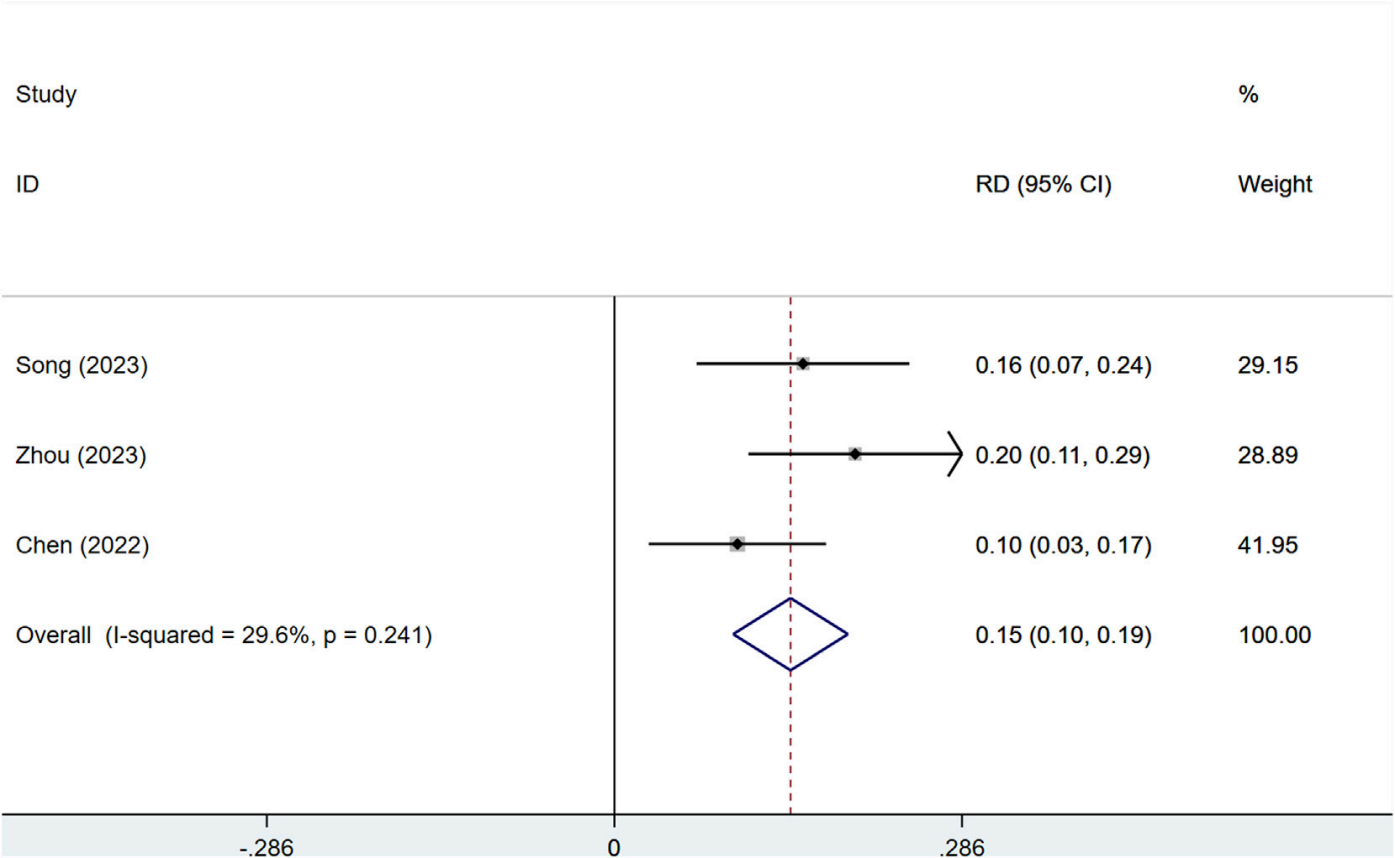
Forest plot of the meta-analysis of DCR in RCT studies.

With low heterogeneity (I^2^ = 7%, P = 0.341), a fixed-effects model was utilized. Serplulimab notably improved DCR in patients with solid tumors [RD = 0.04, 95% CI (0.01–0.07), P < 0.01], as depicted in Figure 4. The assessment of publication bias showed a P value >0.05, indicating a reduced chance of publication bias, as presented in Supplementary Figure S6. The combined analysis of three results showed that the absolute difference in DCR of Serplulimab in combination with chemotherapy ranged from 2.6% to 8.3%, suggesting that it has a consistent effect of remission rate enhancement in a variety of solid tumors.

#### 3.3.3 Progression-free survival (PFS)

**FIGURE 5:**
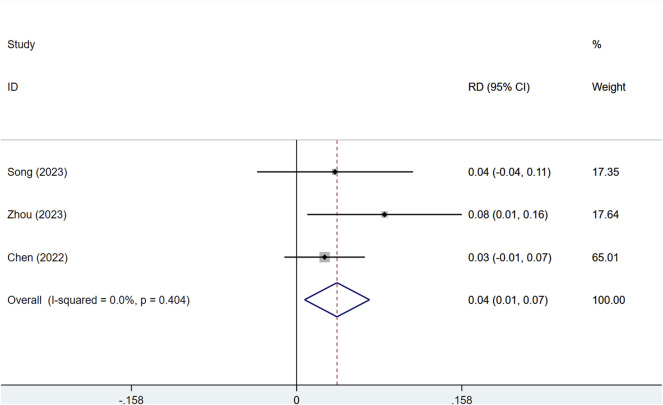
Forest plot of the meta-analysis of PFS in RCT studies.

Three clinical studies provided information on the PFS of patients. Owing to no heterogeneity (I^2^ = 0%, P = 0.376), a fixed-effects approach was utilized. The serplulimab group considerably enhanced PFS in patients [HR = 0.53, 95% CI (0.47–0.61), P < 0.01], as depicted in Figure 5 A P-value >0.05 was observed in Egger’s test, indicating a reduced chance of publication bias, as shown in Supplementary Figure S2. All three studies showed that serplulimab in combination with chemotherapy significantly prolonged PFS in patients, with an absolute median PFS prolongation ranging from 0.5–2.6 months.

#### 3.3.4 Overall survival (OS)

**FIGURE 6:**
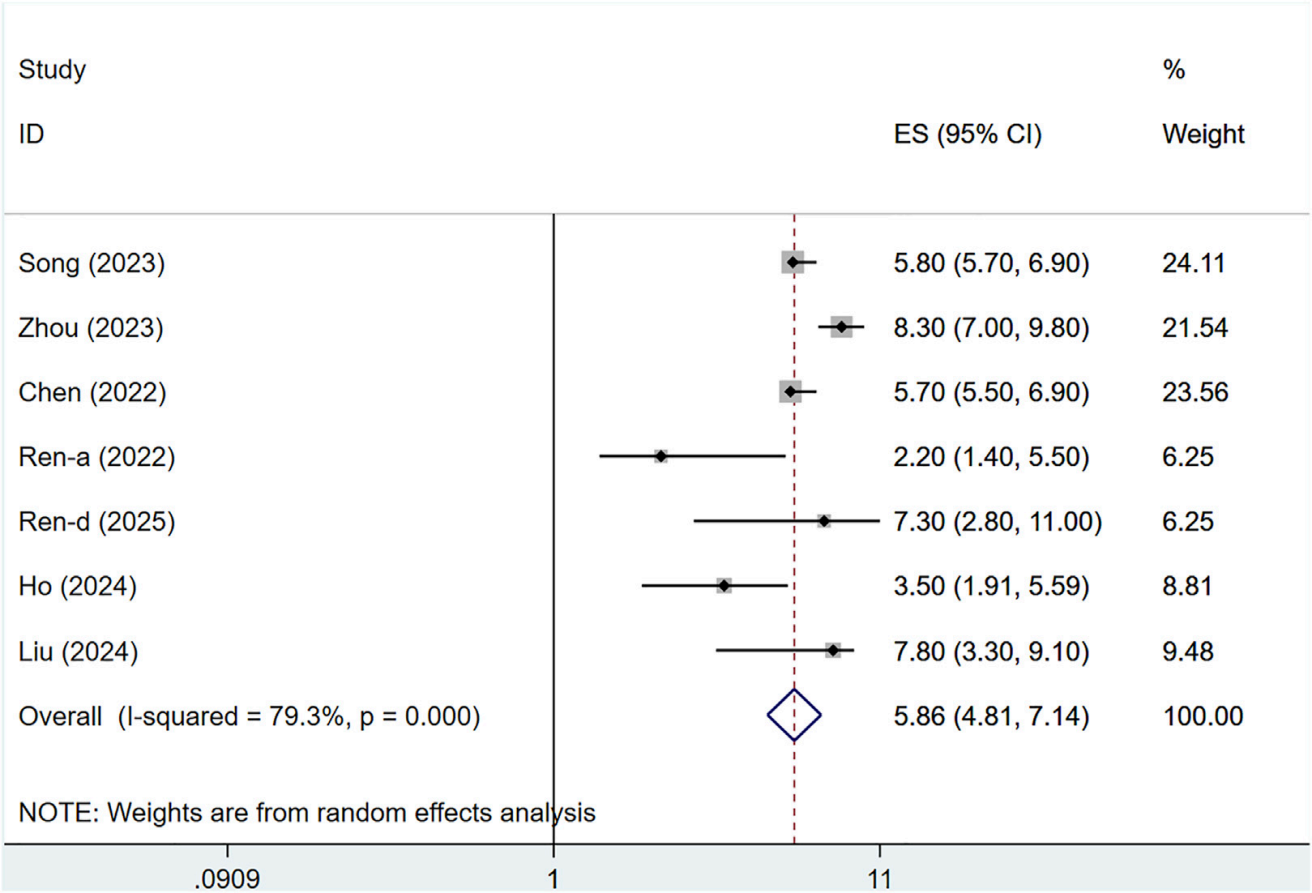
Forest plot of the meta-analysis of OS in RCT studies.

Three clinical studies provided information on the OS of patients. Because of no heterogeneity (I^2^ = 0%, P = 0.710), a fixed effects approach was utilized. Serplulimab profoundly enhanced OS in patients [HR = 0.68, 95% CI (0.59–0.79), P < 0.01], as depicted in Figure 6 A P-value of 0.049 was observed in Egger’s test, indicating a high likelihood of publication bias, as shown in Supplementary Figure S1. All three studies showed that serplulimab in combination with chemotherapy significantly prolonged OS in patients, with an absolute median OS prolongation ranging from 3.5–4.5 months.

### 3.4 Meta-analysis results of single-arm studies

#### 3.4.1 Objective response rate (ORR)

**FIGURE 7:**
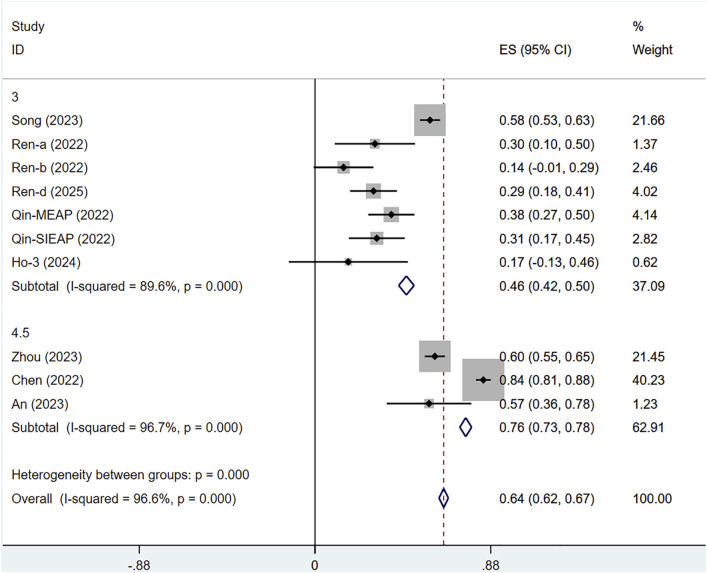
Forest plot of ORR in single-arm studies.

Ten studies assessed the ORR of 1,462 patients with solid tumors. There was substantial heterogeneity across the studies (I^2^ = 96.9%, P < 0.001), and a random-effects approach was utilized. The ORR for serplulimab in solid tumor patients was [ES = 45%, 95% CI (31%–59%), P < 0.01], as illustrated in Figure 7. Sensitivity analysis indicated minimal sensitivity and stable results, as presented in Supplementary Figure S2. A P-value of 0.012 was observed in Egger’s test, indicating the presence of publication bias, as shown in Supplementary Figure S5.

#### 3.4.2 Disease control rate (DCR)

**FIGURE 8:**
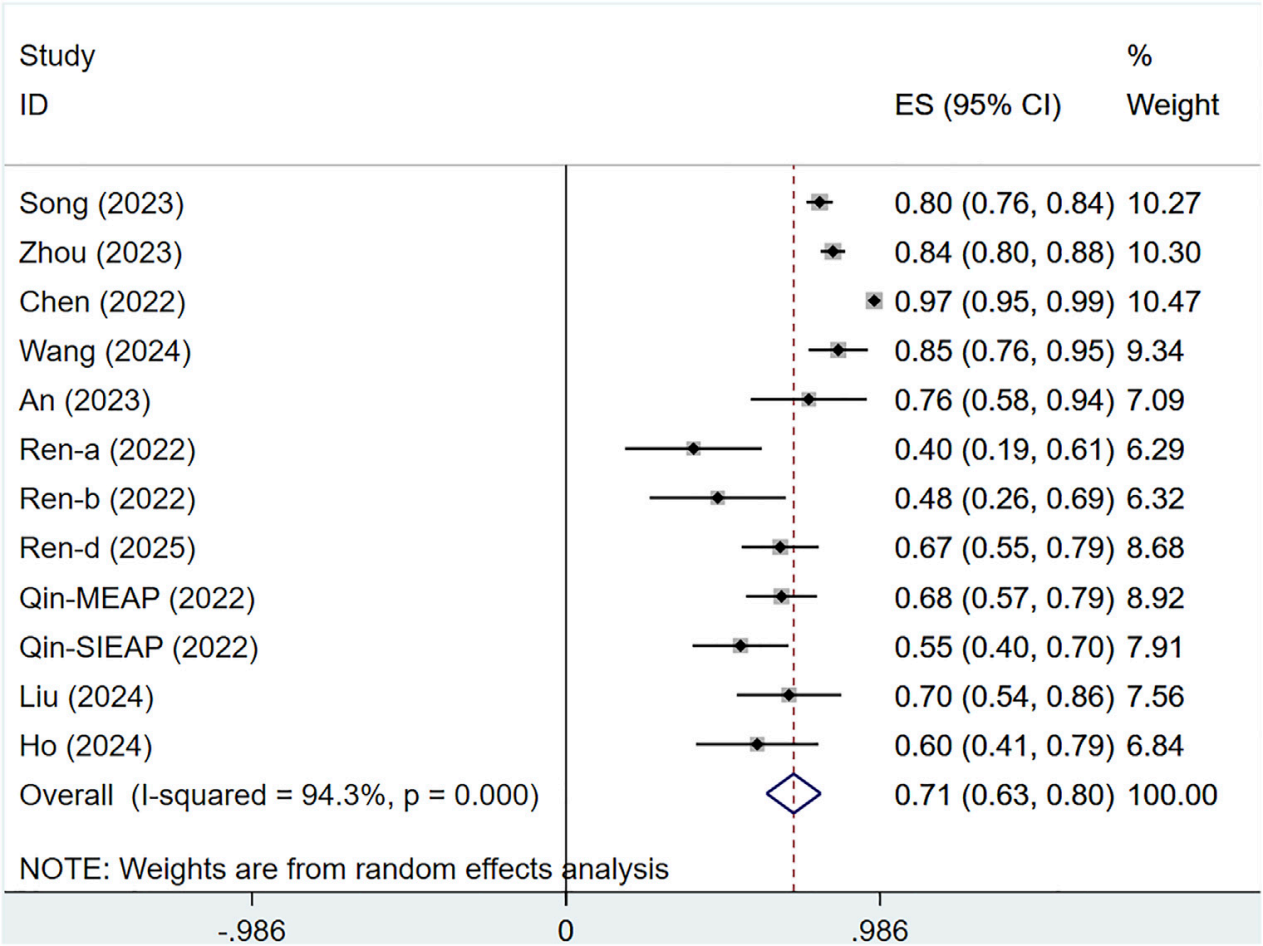
Forest plot of DCR in single-arm studies.

Nine studies evaluated the DCR of 1,462 patients with solid tumors. Marked heterogeneity was evident across the studies (I^2^ = 94.3%, P < 0.001), and a random-effects approach was utilized. The DCR for serplulimab in solid tumor patients was [ES = 71%, 95% CI (63%–80%), P = 0.00], as depicted in Figure 8. Sensitivity analysis indicated minimal sensitivity and stable results, as presented in Supplementary Figure S3. A P-value<0.001 was observed in Egger’s test, indicating the presence of publication bias, as presented in Supplementary Figure S7.

#### 3.4.3 Progression-free survival (PFS)

**FIGURE 9:**
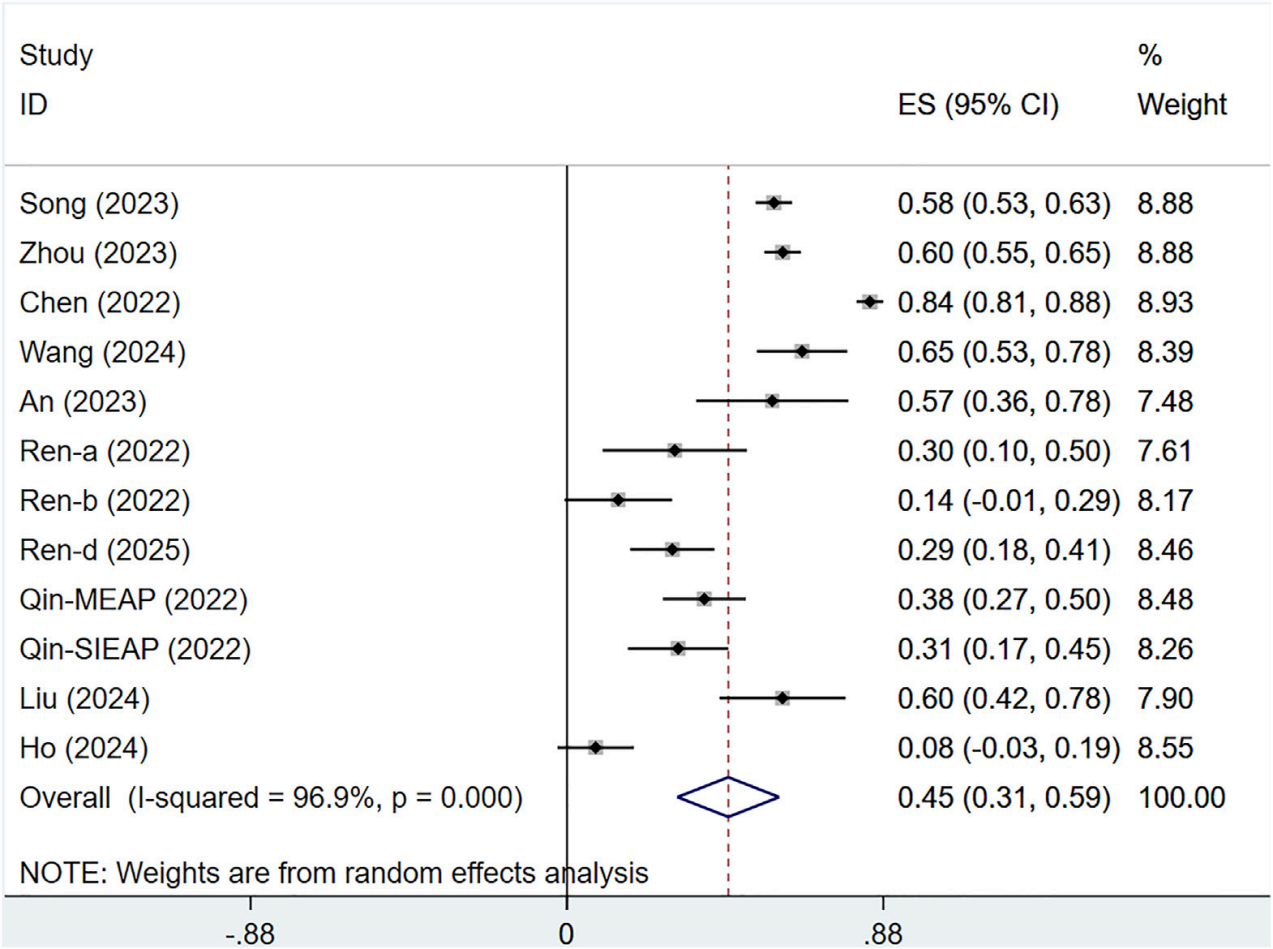
Forest plot of PFS in single-arm studies.

Seven clinical studies reported PFS data for 1,255 patients. Owing to substantial heterogeneity (I^2^ = 79.3%, P < 0.001), a random-effects approach was utilized. The median PFS for serplulimab-treated patients with solid tumors was 5.86 months (95% CI: 4.81–7.14), as illustrated in Figure 9. Sensitivity analysis indicated minimal sensitivity, with stable results, as presented in Supplementary Figure S4. A P-value >0.05 was observed in Egger’s test for publication bias, indicating a reduced chance of publication bias, as shown in Supplementary Figure S3.

### 3.5 Subgroup analysis

#### 3.5.1 Subgroup analysis by tumor types

**FIGURE 10:**
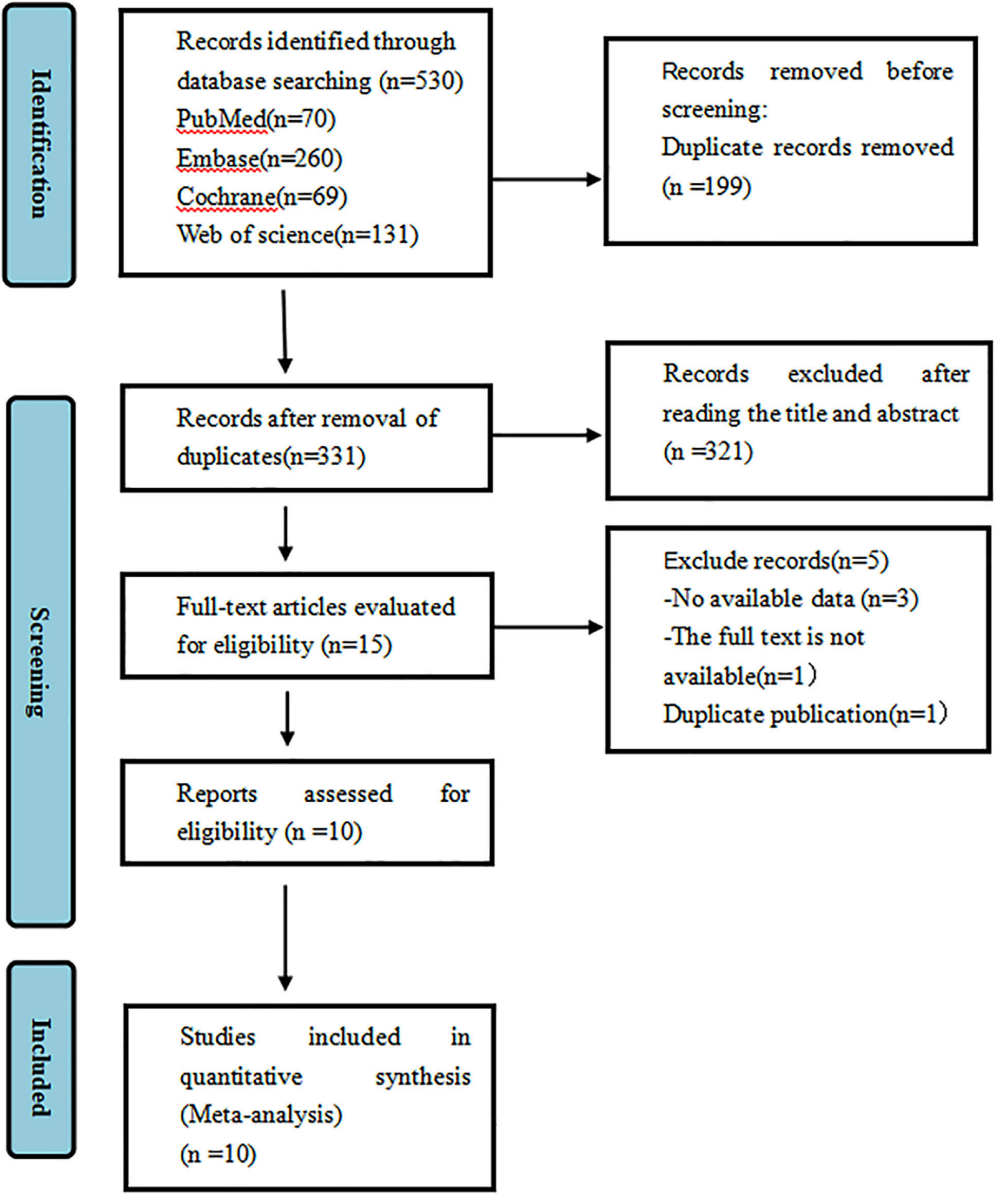
Subgroup analysis by tumor types.

A subgroup analysis by tumor type revealed that heterogeneity could not be resolved. The ORR for serplulimab treatment of various cancers was ORR = 40% [95% CI (22%–58%); P < 0.001] for gastrointestinal tumors, ORR = 72% [95% CI (48%–96%); P < 0.01] for lung cancer, ORR = 57% [95% CI (36%–78%); P < 0.01] for cervical cancer, ORR = 26% [95% CI (6%–45%); P < 0.01] for solid tumors, as presented in Figure 10.

#### 3.5.2 Subgroup analysis by serplulimab dose

**FIGURE 11:**
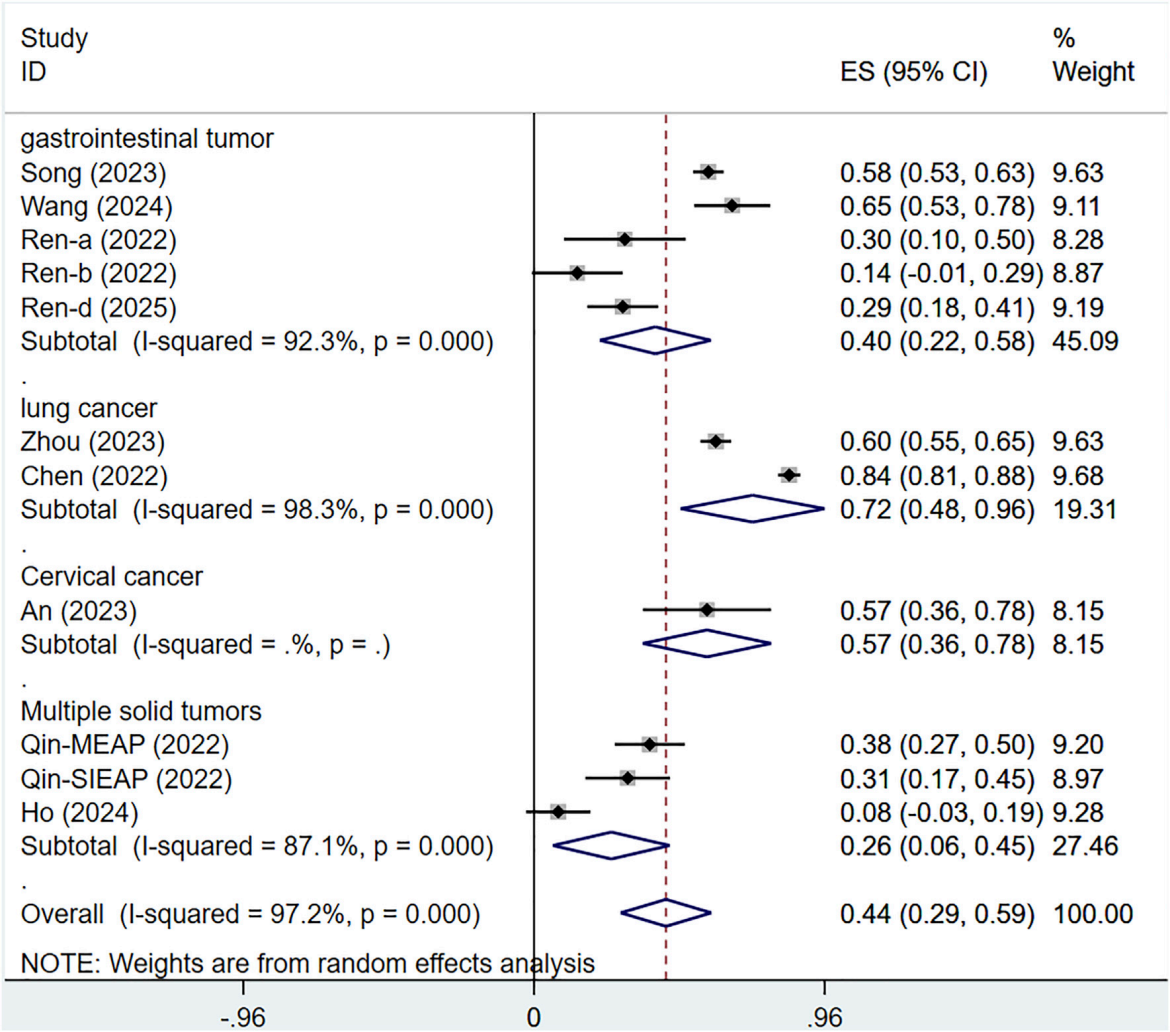
Subgroup analysis by does.

Among the 10 included studies, we conducted a subgroup analysis based on the dosage of serplulimab. Due to inconsistencies in dosage units, the studies by Wang 2024 and Liu 2024 were excluded. Extreme dosage groups (0.3 mg/kg, 1 mg/kg, and 10 mg/kg) were all derived from Ho 2024. After careful review and analysis, we excluded these extreme dosages and retained only the 3 mg/kg dosage group from the study by Ho 2024. The subgroup analysis revealed that the efficacy of serplulimab was dose-dependent, and 4.5 mg/kg serplulimab demonstrated significantly higher ORR than 3 mg/kg in most tumors. Specifically, the ORR for 3 mg/kg serplulimab in solid tumor patients was [ES = 46%, 95% CI (42%–50%); P < 0.01], while the ORR for 4.5 mg/kg was [ES = 76%, 95% CI (73%–78%); P < 0.01], as presented in Figure 11.

### 3.6 Sensitivity analysis

We employed meta-regression to investigate the sources of heterogeneity by constructing regression models incorporating study type, Serplulimab dosage, publication year, and study design to quantify their contributions to heterogeneity. For the dosage regression analysis, studies by Wang 2024 and Liu 2024 were excluded due to inconsistent dosage units. The extreme dosage groups (0.3 mg/kg, 1 mg/kg, and 10 mg/kg) were all derived from the study by Ho 2024. After thorough review and analysis, these extreme dosages were excluded, and only the 3 mg/kg dosage group from the study by Ho 2024 was retained.

The meta-regression results demonstrated that publication year and tumor type had no significant impact on ORR. An increased dosage was positively correlated with the ESs of ORR (P = 0.01). Different study designs (RCT vs single-arm study) significantly influenced the results (P = 0.001). These details are summarized in Table 3.

**TABLE 3:** Meta-regression results.

Outcome	Variate	Coef	Std. Err	P>|t|	[95% conf. Interval]
Orr	Tumor Type	−0.315	0.238	0.222	(-0.865,0.233)
	Dose	0.238	0.071	0.01	(0.074,0.401)
	Year	−0.101	0.791	0.882	(-0.167,0.146)
	Study design	0.308	0.064	0.001	(0.220,0.510)

### 3.7 Meta-analysis for adverse events

In the ten included studies, the main adverse events associated with serplulimab were increased alanine aminotransferase, vomiting, anemia, decreased neutrophil count, decreased appetite, increased aspartate aminotransferase, asthenia, increased blood bilirubin, constipation, diarrhea, decreased lymphocyte count, elevated γ-glutamyltransferase, hyperthyroidism, hypokalemia, hyponatremia, hypothyroidism, nausea, decreased platelet count, pneumonia, proteinuria, pruritus, pyrexia, rash, thrombocytopenia, and weight loss, as well as decreased WBC count.

For any grade adverse events, the ESs were as follows: anemia = 29%, decreased appetite = 29%, increased aspartate aminotransferase = 26%, asthenia = 24%, nausea = 27%, decreased neutrophil count = 26%, decreased neutrophil count = 15%, decreased platelet count = 33%, proteinuria = 28%, vomiting = 21%, weight loss = 21%, WBC count = 30%. For grade ≥3 adverse events, the ESs were: anemia = 8%, hyponatremia = 4%, decreased lymphocyte count = 3%, pneumonia = 2%, thrombocytopenia = 2%, decreased WBC count = 8%, as exhibited in SM 4.

## 4 Discussion

We believe this is the first meta-analysis available exploring the effectiveness and safety of serplulimab in treating solid tumors. Our study results imply that serplulimab may be effective for treating solid tumors based on the current research findings.

Immunotherapy, exemplified by ICIs, has markedly enhanced the treatment outcomes for many cancer patients, and more treatment options are continuously being explored. The Chinese Society of Clinical Oncology (CSCO) has updated the Clinical Guidelines for the Application of Immune Checkpoint Inhibitors in 2024, which includes recommendations for serplulimab in treating (NSCLC), extensive-stage (SCLC), esophageal cancer, and dMMR/MSI-H solid tumors (Fu et al., 2024; Committee CA-cAETIRP, 2024; Kong et al., 2023). The effects of serplulimab vary across different types of solid tumors (Qin et al., 2022; Ho et al., 2024). This research compares the effectiveness and safety of serplulimab across various solid tumors.

The analysis of three RCTs demonstrated that serplulimab significantly improved the ORR, DCR, OS, and PFS in solid tumor patients. In preclinical studies, its predecessor, HLX10, has been established to have antitumor activity in multiple mouse cancer models (Issafras et al., 2021), including homologous breast cancer EMT-6 and colon cancer MC38 models, as well as two tumor/human peripheral blood mononuclear cell (hPBMC) co-culture cancer models. In the EMT-6 model, HLX10 showed a tumor growth inhibition rate (TGI) of up to 51%, in the MC38 model it showed a TGI of more than 80%, and in the HT-29/hPBMC model for colorectal cancer, it demonstrated a TGI of up to 53%. In the (NSCLC), NCI-H292/hPBMC model, it showed a TGI of 60%. Li et al. (Li et al., 2023) compared the efficacy of six different PD-1/PD-L1 inhibitors, either alone or in combination with chemotherapy, in the treatment of extensive-stage small cell lung cancer, including PD-L1 inhibitor (atezolizumab or durvalumab or adebrelimab) plus chemotherapy *versus* chemotherapy alone, and PD-1 inhibitor (pembrolizumab or nivolumab or serplulimab) plus chemotherapy *versus* chemotherapy alone. The study found that serplulimab, when administered with chemotherapy provided the best ORR (OR = 1.7, 95% CI: 1.15–2.53), OS (HR = 0.63, 95% CI: 0.49–0.82), and PFS (HR = 0.47, 95% CI: 0.38–0.59). Overall, serplulimab combined with chemotherapy showed significantly better efficacy than other drugs, ranking first in all PD-1/PD-L1 inhibitor combinations for OS, PFS, and ORR. Moreover, Lu et al. (2023) compared seven different PD-1 inhibitors in the therapy of ESCC. The results highlighted that PD-1 inhibitors, including serplulimab, among others, outperformed standard chemotherapy in OS, PFS, ORR, and DOR across all populations, including first-line and second-line treatment groups, as well as immunotherapy and immunochemotherapy groups.

For single-arm studies, serplulimab showed an ORR of 45% [ES = 45%, 95% CI (31%–59%); P = 0.00] and a DCR of 72% [ES = 72%, 95% CI (62%–80%); P = 0.00]. Our result mirrors the findings of Qin et al. (2022), where serplulimab was used to treat MSI-H/dMMR tumors masses, including gastric, endometrial, and colorectal cancers, with a major efficacy group ORR of 38.2% and a DCR of 67.6%. In a Phase II clinical trial (Marabelle et al., 2020), Marabelle et al. studied the efficacy of pembrolizumab in treating 27 types of MSI-H/dMMR solid tumors, including pancreatic cancer, gastric cancer, bile duct cancer, and endometrial cancer, with an ORR of 34.3%, slightly lower than that of serplulimab.Furthermore, Nivolumab provides clinical benefit (objective response rate [ORR], 31%; 95% CI, 20.8–42.9) in previously treated patients with DNA mismatch repair-deficient (dMMR)/microsatellite instability-high (MSI-H) metastatic colorectal cancer (mCRC) (Overman et al., 2018),also slightly lower than that of serplulimab.

The risk of adverse events (AEs) reported for serplulimab was similar to those for pembrolizumab and nivolumab (Khoja et al., 2017; Baxi et al., 2018; Wang et al., 2020). According to a meta-analysis (Zhang et al., 2024) on the effectiveness and safety of pembrolizumab, the three most commonly reported AEs were diarrhea, anemia, and nausea, with incidences of 0.25 (95% CI: 0.09–0.41), 0.25 (95% CI: 0.00–0.61), and 0.21 (95% CI: 0.06–0.36), respectively. The incidences of anemia and nausea were similar to those seen with serplulimab, which had incidences of 0.29 (95% CI: 0.09–0.48) and 0.27 (95% CI: 0.08–0.45), respectively. The four most common adverse reactions associated with serplulimab were hematological, including decreased white blood cell count [0.30 (95% CI: 0.17–0.44)], decreased platelet count [0.32 (95% CI: 0.20–0.43)], decreased neutrophil count [0.26 (95% CI: 0.13–0.40)], and anemia [0.29 (95% CI: 0.09–0.48)].

Serplulimab, as a novel fully humanized IgG4 monoclonal antibody, demonstrates significant advantages in reducing immunogenicity and toxicity while enhancing clinical efficacy. Its light chain (VL) and heavy chain (VH) utilize humanized CDR grafting (derived from murine antibody 1G4) with framework regions (FR) based on human germline genes (IGKV1-39*01 and IGHV3-11*04), which substantially minimizes heterogenicity and avoids being recognized by the immune system as a foreign antigen (Kong et al., 2023).

The IgG4 Fc region inherently does not strongly binds to Fcγ receptors (e.g., CD16) and complement C1q, thereby avoiding antibody-dependent cellular cytotoxicity (ADCC) and complement-dependent cytotoxicity (CDC) effects. This ensures targeted therapy by preventing the depletion of PD-1-positive T cells. Additionally, the substitution of serine (S) at position 228 in the IgG4 hinge region with proline (P) stabilizes the hinge, preventing unanticipated Fab-arm exchange and the formation of bispecific antibodies, thereby improving both efficacy and safety (Issafras et al., 2021).

Furthermore, the IgG4 subclass design of serplulimab may extend half-life. The CH2-CH3 domains of IgG4 retain natural FcRn binding sites, enabling efficient recycling through FcRn. In acidic environments (e.g., endosomal pH 6.0), the Fc region binds to FcRn, protecting the antibody from lysosomal degradation, and is subsequently released back into circulation at physiological pH (7.4). This extends its plasma half-life to 15–20 days (Tam et al., 2017). The S228P mutation further stabilizes the disulfide bonds in the hinge region, maintaining monomeric integrity and reducing *in vivo* degradation, thereby prolonging its half-life. In a pharmacokinetic study conducted by Issafras et al. (2021), serplulimab exhibited a long half-life (137.97–256.99 h) in cynomolgus monkeys, with an even further extended half-life observed in humans. Therefore, the frequency of administering serplulimab in clinical practice is reduced significantly improving patient compliance.

The study is subject to the following limitations. First, the number of studies included is restricted, and RCTs are relatively rare, which could affect the conclusions. However, serplulimab significantly increased antitumor activity across different tumor types. Second, the included tumor types varied, leading to significant heterogeneity, which could not be resolved even after subgroup analyses. Third, there is potential publication bias. In this study, Egger’s test indicated potential publication bias in certain outcomes (e.g., ORR and DCR in single-arm studies), suggesting that included studies may have preferentially reported positive results for serplulimab, while negative or neutral findings might have been overlooked or unpublished. Due to the limited number of RCTs (only 3 RCTs) and predominance of single-arm studies, there is a further increased risk of selective reporting.

## 5 Conclusion

Serplulimab has demonstrated significant antitumor activity across various solid tumors. However, due to the presence of selection bias in single-arm studies, additional RCTs are necessary to substantiate our findings.

## Data Availability

The original contributions presented in the study are included in the article/Supplementary Material, further inquiries can be directed to the corresponding author.
