# Supplementary material for: Efficacy and safety of serplulimab in solid tumors: a meta-analysis
Source: Front Pharmacol. 2025 Jun 18;16:1604874. doi: 10.3389/fphar.2025.1604874 (PMC12213645; doi:10.3389/fphar.2025.1604874)
Supplement: Supplementary file 1 [file Supplementaryfile1.docx]

**Supplementary material 1** Search strategy

1. Cochrane search history

Search Name:

Date Run: 05/05/2025 22:36:46

Comment:

ID Search Hits

#1 (serplulimab):ti,ab,kw OR (hansizhuang):ti,ab,kw OR (hlx 10):ti,ab,kw OR (hlx10):ti,ab,kw 70

#2 MeSH descriptor: [Neoplasms] explode all trees 127284

#3 (Neoplasms):ti,ab,kw OR (Tumor):ti,ab,kw OR (Neoplasm):ti,ab,kw OR (Tumors):ti,ab,kw OR ("neoplasia"):ti,ab,kw 183532

#4 (Neoplasias):ti,ab,kw OR (Cancer):ti,ab,kw OR (Cancers):ti,ab,kw OR (Malignant Neoplasm):ti,ab,kw OR (Malignancy):ti,ab,kw 224634

#5 (Malignant Neoplasms):ti,ab,kw OR (Neoplasm, Malignant):ti,ab,kw OR (Neoplasms, Malignant):ti,ab,kw OR (Benign Neoplasms):ti,ab,kw OR (Benign Neoplasm):ti,ab,kw 15218

#6 (Neoplasms, Benign):ti,ab,kw OR (Neoplasm, Benign):ti,ab,kw 4010

#7 #2 or #3 or #4 or #5 or #6 290391

#8 #1 and #7 69

2.Embase search history

| No. | Query | Results | Date |
| --- | --- | --- | --- |
| #27 | #6 AND #26 | 260 | 5-May-25 |
| #26 | #7 OR #8 OR #9 OR #10 OR #11 OR #12 OR #13 OR #14 OR #15 OR #16 OR #17 OR #18 OR #19 OR #20 OR #21 OR #22 OR #23 OR #24 OR #25 | 7595057 | 5-May-25 |
| #25 | 'neoplasm, benign':ab,ti | 99 | 5-May-25 |
| #24 | 'neoplasms, benign':ab,ti | 185 | 5-May-25 |
| #23 | 'benign neoplasm':ab,ti | 3017 | 5-May-25 |
| #22 | 'benign neoplasms':ab,ti | 3163 | 5-May-25 |
| #21 | 'neoplasms, malignant':ab,ti | 104 | 5-May-25 |
| #20 | 'neoplasm, malignant':ab,ti | 80 | 5-May-25 |
| #19 | 'malignant neoplasms':ab,ti | 15289 | 5-May-25 |
| #18 | 'malignancies':ab,ti | 262924 | 5-May-25 |
| #17 | 'malignancy':ab,ti | 316120 | 5-May-25 |
| #16 | 'malignant neoplasm':ab,ti | 9327 | 5-May-25 |
| #15 | 'cancers':ab,ti | 543425 | 5-May-25 |
| #14 | 'cancer':ab,ti | 3414371 | 5-May-25 |
| #13 | 'neoplasias':ab,ti | 8650 | 5-May-25 |
| #12 | 'neoplasia':ab,ti | 92403 | 5-May-25 |
| #11 | 'tumors':ab,ti | 1088027 | 5-May-25 |
| #10 | 'neoplasm':ab,ti | 108566 | 5-May-25 |
| #9 | 'tumor':ab,ti | 2163139 | 5-May-25 |
| #8 | 'neoplasms':ab,ti | 154990 | 5-May-25 |
| #7 | 'neoplasm'/exp | 6621278 | 5-May-25 |
| #6 | #1 OR #2 OR #3 OR #4 OR #5 | 266 | 5-May-25 |
| #5 | 'hlx10':ab,ti | 7 | 5-May-25 |
| #4 | 'hlx 10':ab,ti | 0 | 5-May-25 |
| #3 | 'hansizhuang':ab,ti | 0 | 5-May-25 |
| #2 | 'serplulimab':ab,ti | 128 | 5-May-25 |
| #1 | 'serplulimab'/exp | 258 | 5-May-25 |

3.Pubmed search history

| Search number | Query | Sort By | Filters | Search Details | Results | Time |
| --- | --- | --- | --- | --- | --- | --- |
| 5 | ((((serplulimab[Title/Abstract]) OR (hansizhuang[Title/Abstract])) OR (hlx 10[Title/Abstract])) OR (hlx10[Title/Abstract])) AND ((Neoplasms[MeSH Terms]) OR ((((((((((((((((((Neoplasms[Title/Abstract]) OR (Tumor[Title/Abstract])) OR (Neoplasm[Title/Abstract])) OR (Tumors[Title/Abstract])) OR (Neoplasia[Title/Abstract])) OR (Neoplasias[Title/Abstract])) OR (Cancer[Title/Abstract])) OR (Cancers[Title/Abstract])) OR (Malignant Neoplasm[Title/Abstract])) OR (Malignancy[Title/Abstract])) OR (Malignancies[Title/Abstract])) OR (Malignant Neoplasms[Title/Abstract])) OR (Neoplasm, Malignant[Title/Abstract])) OR (Neoplasms, Malignant[Title/Abstract])) OR (Benign Neoplasms[Title/Abstract])) OR (Benign Neoplasm[Title/Abstract])) OR (Neoplasms, Benign[Title/Abstract])) OR (Neoplasm, Benign[Title/Abstract]))) |  |  | ("serplulimab"[Title/Abstract] OR ("hlx"[All Fields] AND "10"[Title/Abstract]) OR "hlx10"[Title/Abstract]) AND ("Neoplasms"[MeSH Terms] OR ("Neoplasms"[Title/Abstract] OR "Tumor"[Title/Abstract] OR "Neoplasm"[Title/Abstract] OR "Tumors"[Title/Abstract] OR "Neoplasia"[Title/Abstract] OR "Neoplasias"[Title/Abstract] OR "Cancer"[Title/Abstract] OR "Cancers"[Title/Abstract] OR "malignant neoplasm"[Title/Abstract] OR "Malignancy"[Title/Abstract] OR "Malignancies"[Title/Abstract] OR "malignant neoplasms"[Title/Abstract] OR "neoplasm malignant"[Title/Abstract] OR "neoplasms malignant"[Title/Abstract] OR "benign neoplasms"[Title/Abstract] OR "benign neoplasm"[Title/Abstract] OR "neoplasms benign"[Title/Abstract] OR "neoplasm benign"[Title/Abstract])) | 70 | 10:32:06 |
| 4 | (Neoplasms[MeSH Terms]) OR ((((((((((((((((((Neoplasms[Title/Abstract]) OR (Tumor[Title/Abstract])) OR (Neoplasm[Title/Abstract])) OR (Tumors[Title/Abstract])) OR (Neoplasia[Title/Abstract])) OR (Neoplasias[Title/Abstract])) OR (Cancer[Title/Abstract])) OR (Cancers[Title/Abstract])) OR (Malignant Neoplasm[Title/Abstract])) OR (Malignancy[Title/Abstract])) OR (Malignancies[Title/Abstract])) OR (Malignant Neoplasms[Title/Abstract])) OR (Neoplasm, Malignant[Title/Abstract])) OR (Neoplasms, Malignant[Title/Abstract])) OR (Benign Neoplasms[Title/Abstract])) OR (Benign Neoplasm[Title/Abstract])) OR (Neoplasms, Benign[Title/Abstract])) OR (Neoplasm, Benign[Title/Abstract])) |  |  | "Neoplasms"[MeSH Terms] OR "Neoplasms"[Title/Abstract] OR "Tumor"[Title/Abstract] OR "Neoplasm"[Title/Abstract] OR "Tumors"[Title/Abstract] OR "Neoplasia"[Title/Abstract] OR "Neoplasias"[Title/Abstract] OR "Cancer"[Title/Abstract] OR "Cancers"[Title/Abstract] OR "malignant neoplasm"[Title/Abstract] OR "Malignancy"[Title/Abstract] OR "Malignancies"[Title/Abstract] OR "malignant neoplasms"[Title/Abstract] OR "neoplasm malignant"[Title/Abstract] OR "neoplasms malignant"[Title/Abstract] OR "benign neoplasms"[Title/Abstract] OR "benign neoplasm"[Title/Abstract] OR "neoplasms benign"[Title/Abstract] OR "neoplasm benign"[Title/Abstract] | 5,320,376 | 10:31:11 |
| 3 | (((((((((((((((((Neoplasms[Title/Abstract]) OR (Tumor[Title/Abstract])) OR (Neoplasm[Title/Abstract])) OR (Tumors[Title/Abstract])) OR (Neoplasia[Title/Abstract])) OR (Neoplasias[Title/Abstract])) OR (Cancer[Title/Abstract])) OR (Cancers[Title/Abstract])) OR (Malignant Neoplasm[Title/Abstract])) OR (Malignancy[Title/Abstract])) OR (Malignancies[Title/Abstract])) OR (Malignant Neoplasms[Title/Abstract])) OR (Neoplasm, Malignant[Title/Abstract])) OR (Neoplasms, Malignant[Title/Abstract])) OR (Benign Neoplasms[Title/Abstract])) OR (Benign Neoplasm[Title/Abstract])) OR (Neoplasms, Benign[Title/Abstract])) OR (Neoplasm, Benign[Title/Abstract]) |  |  | "Neoplasms"[Title/Abstract] OR "Tumor"[Title/Abstract] OR "Neoplasm"[Title/Abstract] OR "Tumors"[Title/Abstract] OR "Neoplasia"[Title/Abstract] OR "Neoplasias"[Title/Abstract] OR "Cancer"[Title/Abstract] OR "Cancers"[Title/Abstract] OR "malignant neoplasm"[Title/Abstract] OR "Malignancy"[Title/Abstract] OR "Malignancies"[Title/Abstract] OR "malignant neoplasms"[Title/Abstract] OR "neoplasm malignant"[Title/Abstract] OR "neoplasms malignant"[Title/Abstract] OR "benign neoplasms"[Title/Abstract] OR "benign neoplasm"[Title/Abstract] OR "neoplasms benign"[Title/Abstract] OR "neoplasm benign"[Title/Abstract] | 3,874,738 | 10:30:23 |
| 2 | Neoplasms[MeSH Terms] |  |  | "neoplasms"[MeSH Terms] | 4,103,243 | 10:27:12 |
| 1 | (((serplulimab[Title/Abstract]) OR (hansizhuang[Title/Abstract])) OR (hlx 10[Title/Abstract])) OR (hlx10[Title/Abstract]) |  |  | "serplulimab"[Title/Abstract] OR ("hlx"[All Fields] AND "10"[Title/Abstract]) OR "hlx10"[Title/Abstract] | 92 | 10:26:48 |

4.Web of science search history

| Entitlements | # | Search Query | Database | Results | Date Run |
| --- | --- | --- | --- | --- | --- |
| - WOS: 1900 to 2025 - BIOSIS: 1994 to 2025 - CSCD: 1989 to 2025 - DIIDW: 1966 to 2025 - FSTA: 1969 to 2025 - GRANTS: 1953 to 2025 - INSPEC: 1898 to 2025 - KJD: 1980 to 2025 - MEDLINE: 1950 to 2025 - PCI: 1950 to 2025 - PPRN: 1991 to 2025 - PQDT: 1637 to 2025 - SCIELO: 2002 to 2025 | 1 | TS=(serplulimab) OR TS=(hansizhuang) OR TS=(hlx 10) OR TS=(hlx10) and Preprint Citation Index (Exclude – Database) | All Databases | 248 | Tue May 06 2025 11:50:06 GMT+0800 (中国标准时间) |
| - WOS: 1900 to 2025 - BIOSIS: 1994 to 2025 - CSCD: 1989 to 2025 - DIIDW: 1966 to 2025 - FSTA: 1969 to 2025 - GRANTS: 1953 to 2025 - INSPEC: 1898 to 2025 - KJD: 1980 to 2025 - MEDLINE: 1950 to 2025 - PCI: 1950 to 2025 - PPRN: 1991 to 2025 - PQDT: 1637 to 2025 - SCIELO: 2002 to 2025 | 2 | TS=(Neoplasms) OR TS=(Tumor) OR TS=(Neoplasm) OR TS=(Tumors) OR TS=(Neoplasia) OR TS=(Neoplasias) OR TS=(Cancer) OR TS=(Cancers) OR TS=(Malignant Neoplasm) OR TS=(Malignancy) OR TS=(Malignancies) OR TS=(Malignant Neoplasms) OR TS=(Neoplasm, Malignant) OR TS=(Neoplasms, Malignant) OR TS=(Benign Neoplasms) OR TS=(Benign Neoplasm) OR TS=(Neoplasms, Benign) OR TS=(Neoplasm, Benign) and Preprint Citation Index (Exclude – Database) | All Databases | 10103324 | Tue May 06 2025 12:00:54 GMT+0800 (中国标准时间) |
| - WOS: 1900 to 2025 - BIOSIS: 1994 to 2025 - CSCD: 1989 to 2025 - DIIDW: 1966 to 2025 - FSTA: 1969 to 2025 - GRANTS: 1953 to 2025 - INSPEC: 1898 to 2025 - KJD: 1980 to 2025 - MEDLINE: 1950 to 2025 - PCI: 1950 to 2025 - PPRN: 1991 to 2025 - PQDT: 1637 to 2025 - SCIELO: 2002 to 2025 | 3 | #1 AND #2 and Preprint Citation Index (Exclude – Database) | All Databases | 131 | Tue May 06 2025 12:02:51 GMT+0800 (中国标准时间) |
