# Supplementary material for: Efficacy and safety of serplulimab in solid tumors: a meta-analysis
Source: Front Pharmacol. 2025 Jun 18;16:1604874. doi: 10.3389/fphar.2025.1604874 (PMC12213645; doi:10.3389/fphar.2025.1604874)
Supplement: Supplementary file 2 [file Supplementaryfile5.docx]

**Supplementary material 5** Excluded Studies Information Table

| Author | Year | Title | Study design | Reason for exclusion |
| --- | --- | --- | --- | --- |
| Huang | 2024 | Rationale and Design of a Phase II Trial of Combined Serplulimab and Chemotherapy in Patients with Histologically Transformed Small Cell Lung Cancer: a Prospective, Single-arm and Multicentre Study | Single-arm | data remain unpublished |
| Xin | 2023 | The efficacy and safety of nanoparticle albumin bound-paclitaxelbased  regimen as second- or third-line treatment in patients with  advanced esophageal squamous cell carcinoma | retrospective study | Duplicate publication |
| Ren | 2023 | A phase II study of serplulimab (a programmed death-1 inhibitor) with or without HLX04 (a bevacizumab biosimilar) for the treatment of advanced hepatocellular carcinoma | **RCT** | Lack of outcome measures(available only as conference abstracts) |
| Xu | 2024 | Phase I/II Clinical Study of PRaG Regimen  Combined With Intraperitoneal Infusion of  PD-1 Inhibitor for Advanced Refractory Solid  Tumors With Cancerous Ascites (PRaG4.0P  Study) | single-arm | data remain unpublished |
| Ma | 2024 | Single-drug Chemotherapy Plus Immunotherapy as First-line Treatment for Stage Ⅳ Non-small Cell Lung Cancer Elderly Patients: A Phase II Clinical Trial UNICORN Study | single-arm | data remain unpublished |
