# Supplementary material for: Efficacy and safety of serplulimab in solid tumors: a meta-analysis
Source: Front Pharmacol. 2025 Jun 18;16:1604874. doi: 10.3389/fphar.2025.1604874 (PMC12213645; doi:10.3389/fphar.2025.1604874)

**Supplementary Material 2** Sensitivity analysis of the meta-analysis

FigureS1. Sensitivity analysis of the meta-analysis of ORR in RCT studies


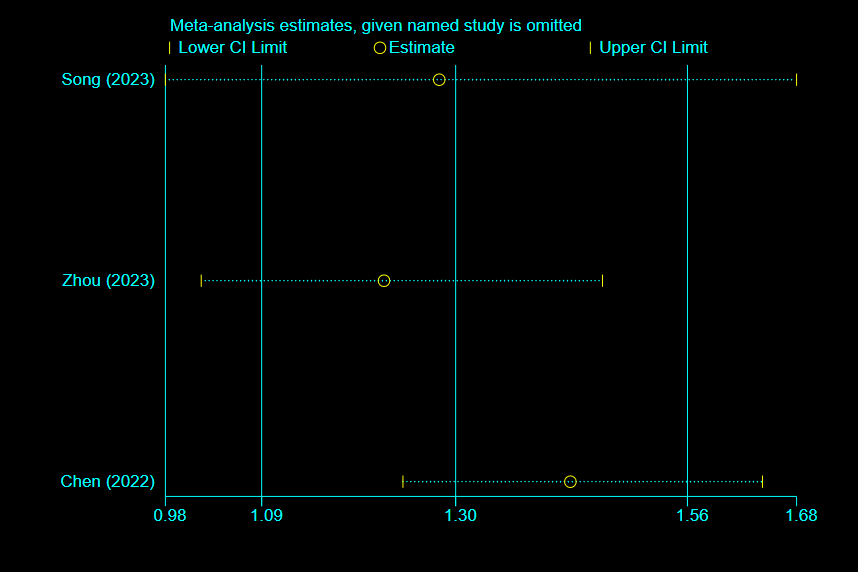


FigureS2.Sensitivity analysis of the meta-analysis of ORR in single-arm studies


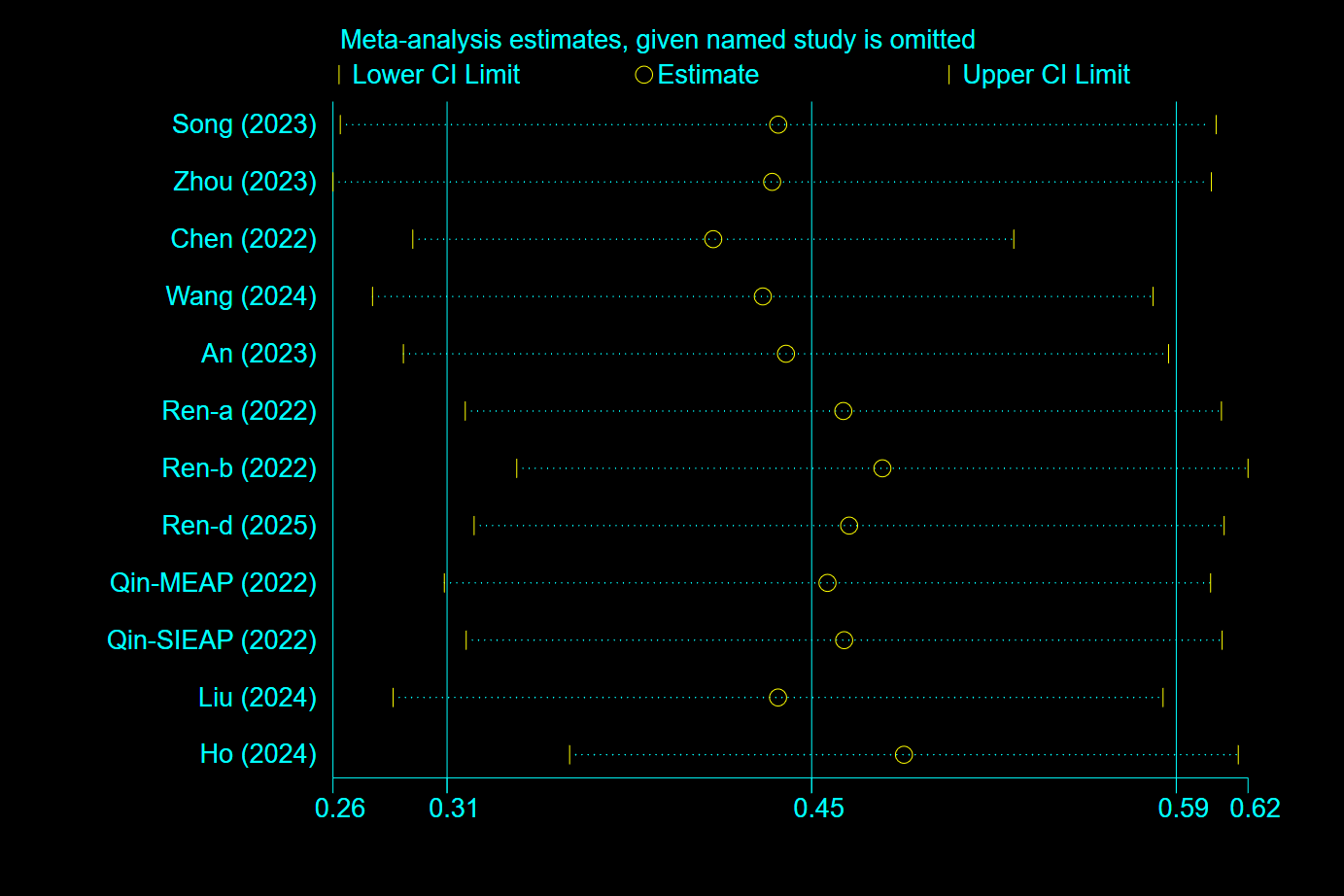


FigureS3.Sensitivity analysis of the meta-analysis of DCR in single-arm studies


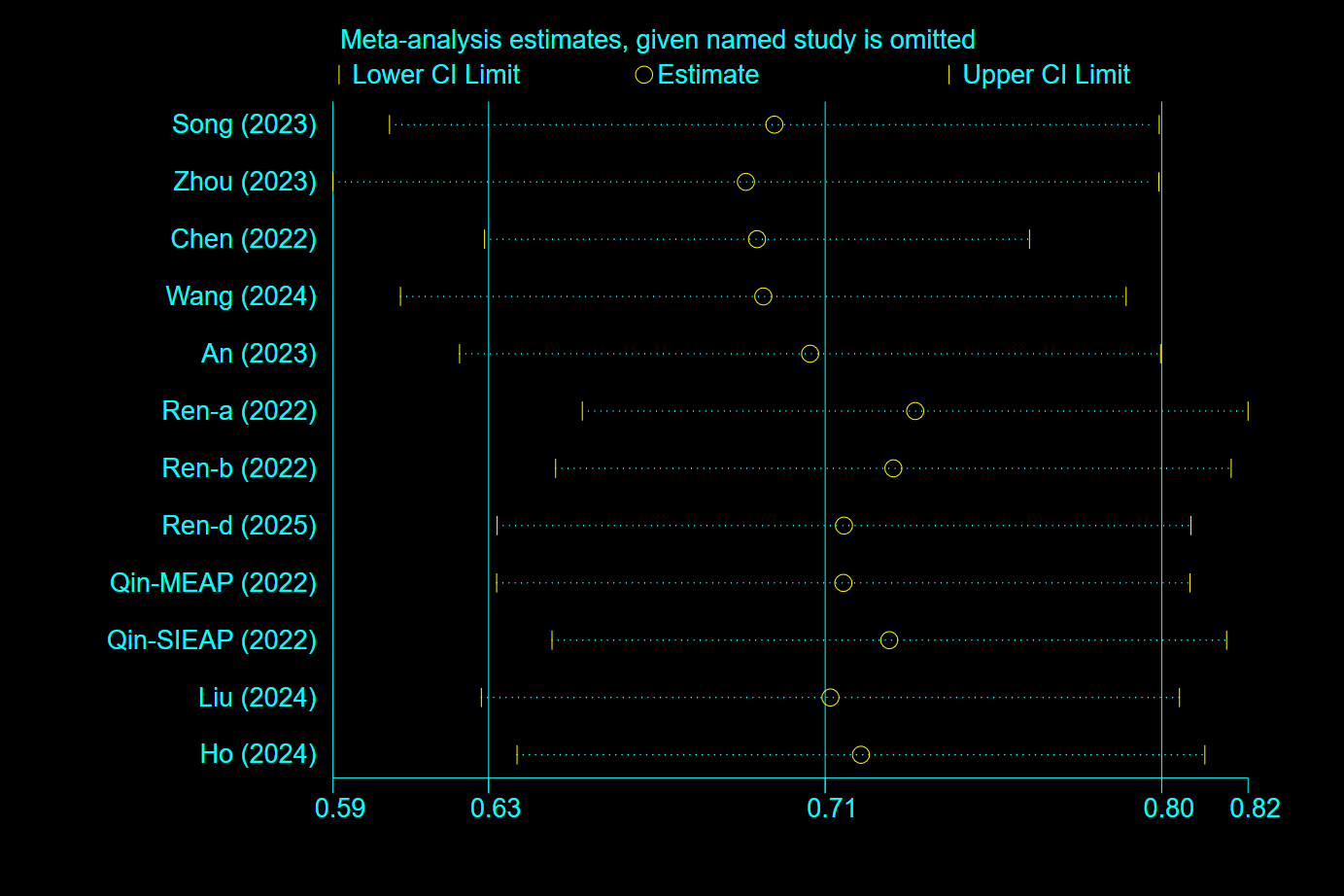


FigureS4. Sensitivity analysis of the meta-analysis of PFS in single-arm studies


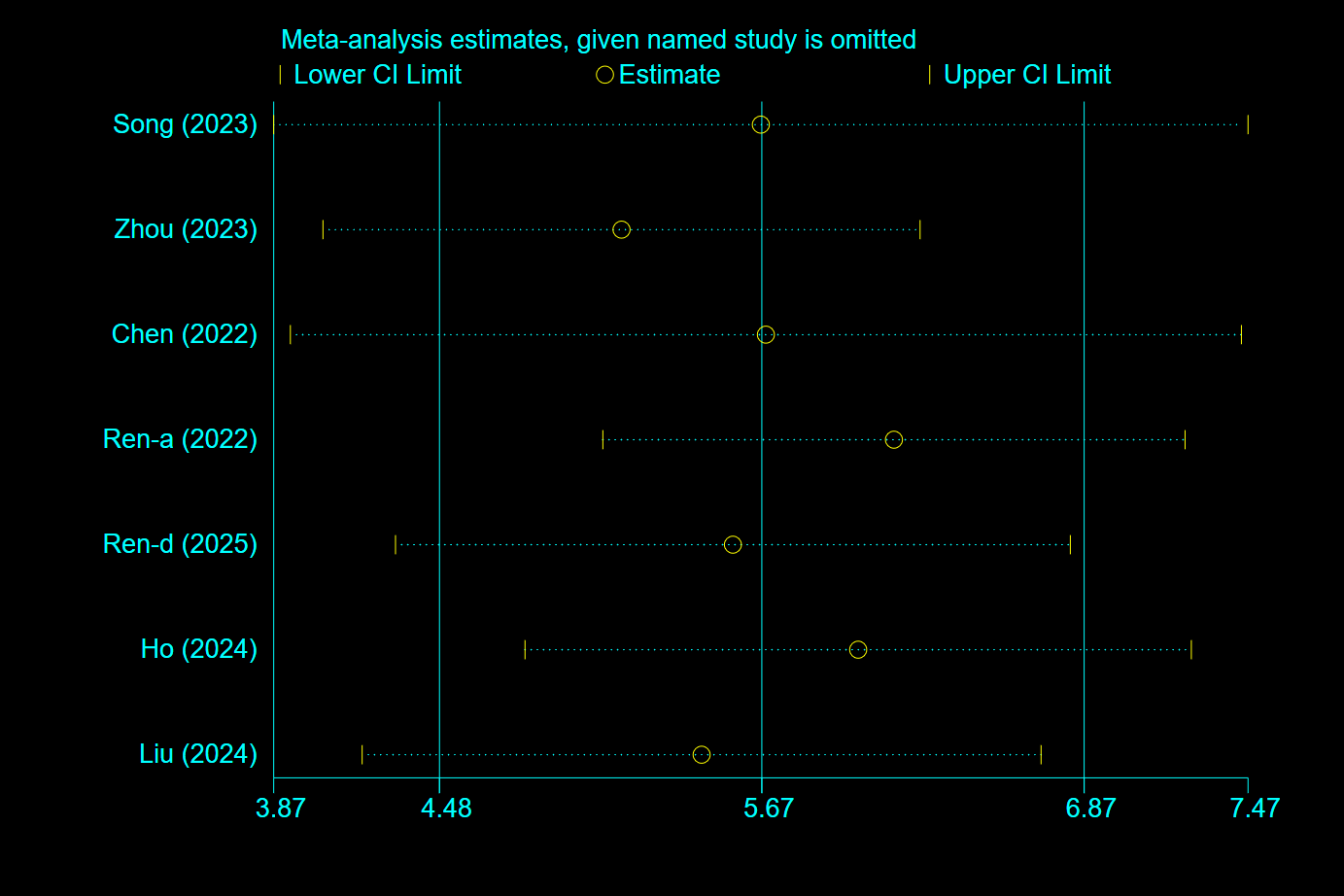

Supplement: Supplementary file 4 [file Supplementaryfile2.docx]
