# Supplementary material for: Efficacy and safety of serplulimab in solid tumors: a meta-analysis
Source: Front Pharmacol. 2025 Jun 18;16:1604874. doi: 10.3389/fphar.2025.1604874 (PMC12213645; doi:10.3389/fphar.2025.1604874)
Supplement: Supplementary file 5 [file Supplementaryfile4.docx]

**Supplementary Material 4** Incidence rate of adverse events

| adverse event | any grade | | | | grade≥3 | | | |
| --- | --- | --- | --- | --- | --- | --- | --- | --- |
|  | study | heterogeneity | | ES（95%CI） | study | heterogeneity | | ES（95%CI） |
|  |  | P | I2(%) |  |  | P | I2(%) |  |
| Abdominal pain upper | 3 | 0.448 | 0.00% | 0.13(0.07,0.19) |  |  |  |  |
| Alanine aminotransferase increased | 6 | 0.001 | 79.90% | 0.18(0.13,0.23) | 5 | 0.559 | 0.00% | 0.01(0.01,0.02) |
| Anemia | 7 | 0 | 98.50% | 0.29(0.09,0.48) | 6 | 0 | 91.00% | 0.08(0.03,0.13) |
| Appetite decreased | 6 | 0 | 93.50% | 0.26(0.13,0.39) |  |  |  |  |
| Aspartate aminotransferase increased | 6 | 0 | 92.30% | 0.25(0.17,0.33) | 5 | 0 | 80.20% | 0.02(-0.00,0.03) |
| Asthenia | 4 | 0 | 93.20% | 0.24(0.11,0.37) | 3 | 0.696 | 0.00% | 0.01(0.00,0.01) |
| Blood bilirubin increased | 4 | 0 | 90.10% | 0.24(0.12,0.35) | 3 | 0.122 | 48.20% | 0.01(0.00,0.02) |
| Constipation | 5 | 0 | 89.20% | 0.17(0.08,0.25) |  |  |  |  |
| Diarrhea | 6 | 0.045 | 53.50% | 0.10(0.07,0.14) |  |  |  |  |
| Elevated γ-glutamyltransferase | 4 | 0.16 | 42.00% | 0.1(0.06,0.14) | 3 | 0.57 | 0.00% | 0.08(0.01,0.15) |
| Fatigue | 3 | 0.022 | 73.70% | 0.1(0.04,0.16) |  |  |  |  |
| Hyperthyroidism | 4 | 0.648 | 0.00% | 0.10(0.08,0.13) |  |  |  |  |
| Hypokalemia | 4 | 0.816 | 0.00% | 0.11(0.08,0.13) | 3 | 0.175 | 42.70% | 0.04(0.02,0.06) |
| Hyponatremia | 4 | 0 | 88.90% | 0.09(0.03,0.15) | 4 | 0.428 | 0.00% | 0.04(0.03,0.06) |
| Hypothyroidism | 8 | 0.007 | 64.30% | 0.11(0.09,0.13) |  |  |  |  |
| Lymphocyte count decreased | 4 | 0.004 | 78.80% | 0.14(0.08,0.20) | 4 | 0.222 | 29.90% | 0.03(0.02,0.04) |
| Nausea | 7 | 0 | 98.40% | 0.27(0.08,0.45) | 3 | 0.013 | 76.90% | 0.01(-0.00,0.02) |
| Neutrophil count decreased | 8 | 0 | 97.20% | 0.26(0.13,0.40) | 5 | 0.022 | 91.80% | 0.13(0.16,0.19) |
| Platelet count decreased | 6 | 0 | 95.00% | 0.32(0.20,0.43) | 5 | 0.329 | 13.40% | 0.06(0.04,0.07) |
| Pneumonia | 4 | 0.413 | 0.00% | 0.06(0.03,0.08) | 3 | 0.646 | 0.00% | 0.02(0.00,0.03) |
| Proteinuria | 5 | 0 | 95.00% | 0.28(0.17,0.38) | 3 | 0.026 | 67.60% | 0.05(-0.01,0.11) |
| Pruritus | 4 | 0.486 | 0.00% | 0.01(0.04,0.16) |  |  |  |  |
| Pyrexia | 6 | 0.032 | 56.40% | 0.12(0.08,0.17) |  |  |  |  |
| Rash | 4 | 0.246 | 27.70% | 0.11(0.08,0.13) |  |  |  |  |
| Thrombocytopenia | 3 | 0.529 | 0.00% | 0.09(0.06,0.12) | 3 | 0.401 | 0.00% | 0.02(0.00,0.03) |
| Vomiting | 6 | 0 | 97.30% | 0.21(0.02,0.40) |  |  |  |  |
| weight decreaesd | 3 | 0.086 | 54.50% | 0.21(0.10,0.31) |  |  |  |  |
| White blood cell count decreased | 8 | 0 | 97.40% | 0.30(0.17,0.44) | 6 | 0 | 80.00% | 0.08(0.04,0.12) |
